# Long term potentiation, but not depression, in interlamellar hippocampus CA1

**DOI:** 10.1038/s41598-018-23369-4

**Published:** 2018-03-26

**Authors:** Duk-gyu Sun, Hyeri Kang, Hannah Tetteh, Junfeng Su, Jihwan Lee, Sung-Won Park, Jufang He, Jihoon Jo, Sungchil Yang, Sunggu Yang

**Affiliations:** 1 Department of Biomedical Sciences, Chonnam National University Medical School, Gwangju, Korea; 2 Department of Nano-bioengineering, Incheon National University, Incheon, Korea; 3 Department of Biomedical Sciences, City University of Hong Kong, Kowloon, Hong Kong; 4 Department of Neurology, Chonnam National University Medical School, Gwangju, Korea; 5 NeuroMedical Convergence Laboratory, Biomedical Research Institute, Chonnam National University Hospital, Gwangju, Korea

## Abstract

Synaptic plasticity in the lamellar CA3 to CA1 circuitry has been extensively studied while interlamellar CA1 to CA1 connections have not yet received much attention. One of our earlier studies demonstrated that axons of CA1 pyramidal neurons project to neighboring CA1 neurons, implicating information transfer along a longitudinal interlamellar network. Still, it remains unclear whether long-term synaptic plasticity is present within this longitudinal CA1 network. Here, we investigate long-term synaptic plasticity between CA1 pyramidal cells, using *in vitro* and *in vivo* extracellular recordings and 3D holography glutamate uncaging. We found that the CA1-CA1 network exhibits NMDA receptor-dependent long-term potentiation (LTP) without direction or layer selectivity. By contrast, we find no significant long-term depression (LTD) under various LTD induction protocols. These results implicate unique synaptic properties in the longitudinal projection suggesting that the interlamellar CA1 network could be a promising structure for hippocampus-related information processing and brain diseases.

## Introduction

Hippocampus anatomy and function are long-standing subjects of plasticity and disease^1–3^. In particular, the lamellar organization of the hippocampal tri-synaptic circuity DG-CA3-CA1 is considered to be a parallel and independent unit^4,5^ and has been a main focus in the investigation of synaptic plasticity. The lamellar perspective of hippocampus has significantly influenced anatomical conceptualization of cognitive functions. Thus, synaptic plasticity and corresponding behavioral changes have been investigated intensely within the lamellar structure^6,7^.

Recently, the neuroscience community recognized that the organization of intrinsic hippocampal connections is more complex than previously thought. Hippocampus is a bilaterally symmetrical and elongated structure, bending in a C-shaped manner from its rostrodorsal septal to caudoventral temporal end (the septo-temporal axis) with fibers coursing through all planes^1^. One study^8^ provided the interlamellar organization of hippocampus is of functional significance. Using *in vivo* hippocampal extracellular recording, the authors demonstrated synchronized activity of interlamellar cells during short-term memory tasks. In addition, other studies propose that CA1 pyramidal cell’s axon collaterals project to neighboring CA1 neurons^4,9,10^. An interlamellar (referred to as associational) network within CA1 has been proposed as a key factor in the development of epilepsy^11^. More recently, the synaptic connections within CA1 associational network along the longitudinal axis of hippocampus has been demonstrated^12^.

While long-lasting, activity-dependent synaptic plasticity such as LTP and LTD in the hippocampal tri-synaptic circuitry has received enormous attention as a cellular correlate of cognitive functions^7,13–17^, longitudinal synaptic plasticity has not been thoroughly investigated so far. Here, we studied long-term synaptic plasticity within the CA1 longitudinal plane using *in vitro* and *in vivo* recordings. We found that the longitudinal network exhibits NMDAR-dependent LTP without direction and layer selectivity. Unexpectedly however, we do not observe significant NMDAR-and mGluR-dependent LTD under frequently used LTD induction protocols. These results implicate a unique anatomical and functional property of the interlamellar projection.

## Materials and Methods

### Animals

Male C57BL/6 J mice were used (5–9 weeks old for *in vitro* slices and 6–12 weeks old for *in vivo* brains). Mice were acclimatized to a 12 h light and dark cycle at 22 ± 2 °C with free access to food and water in a specific pathogen–free facility. All animals were treated in accordance with the guidelines and regulations on Animal Care and Use of Laboratory of NIH, and animal experiments were reviewed and approved by the Institutional Animal Use and Care Committee of Incheon National University (INU-ANIM-2017–08), Chonnam National University (2010–094) and City University of Hong Kong (Ref# 15–90).

### *In vivo* Field Recording

C57BL/6 J mice (6–12 weeks old) were anesthetized with Urethane (0.06 g per 25 g weight) and supplemented with Atropine. After a craniotomy, recording electrodes were placed above the dorsal hippocampus. Stimulation electrodes were placed lateral to the recording electrodes in a longitudinal direction. Electrodes were lowered to hippocampal CA1 layer according to the response of characteristic single unit and evoked postsynaptic potentials. Local field potentials were sampled at 24 kHz and filtered 500 Hz with a Tucker-Davis Technologies system. I-O curve was performed to determine half the maximal stimulus intensity of evoked field excitatory postsynaptic potentials (fEPSPs). LTP was induced with currents eliciting 75% of the maximal EPSP response by high frequency stimulation (HFS, four 100 Hz pulse, 10 s interval); LTD was induced with low frequency stimulation (1 Hz pp-LFS; with or without 50 ms paired-pulse intervals, 900 pairs of stimuli during 15 min). Response change was measured in % of the slope baseline. After recording, 10 μA current was passed through both recording and stimulating electrodes for 30 s to lesion the recorded areas. Then, the mice were trans-cardially perfused with 4% PFA. The brains were harvested, sliced and stained with Cresyl Violet.

### Slice preparation

**Figure 1:**
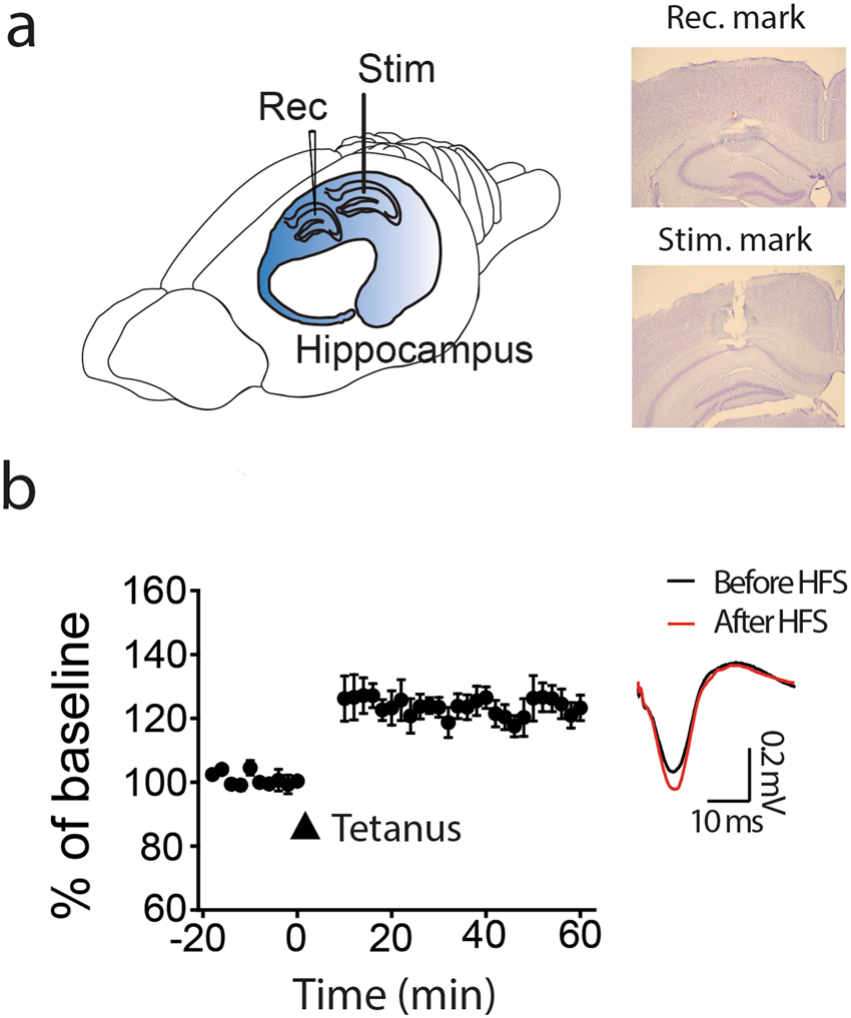
*In vivo* LTP in the interlamellar network. (**a**) A schematic drawing of recording and stimulation electrodes in anesthetized animals. The loci of recording (on the septal side of CA1) and stimulating electrodes (on the temporal side of CA1) were identified by lesion marks. (**b**) LTP is induced in the interlamellar connection by 100 Hz high frequency stimulation (HFS) (n = 10 mice). Color traces: before (black) and after (red) HFS. Error bars represent SE.

The mouse brains were quickly removed and placed into chilled (4 °C), oxygenated (5% CO_2_ and 95% O_2_) slicing medium containing the following ingredients: 212 mM sucrose, 5 mM KCl, 1.23 mM NaH_2_PO_4_, 26 mM NaHCO_3_, 11 mM glucose, 1.5 mM MgCl_2_, and 2.5 mM CaCl_2_. Transverse slices (300–400 μm) were cut orthogonal to the septo-temporal axis of the hippocampus, whereas longitudinal slices (300–400 μm) were cut parallel to the septo-temporal plane (Fig. 1a). The slices were then stored, submerged in aCSF at room temperature for 1–2 h before transferring them to the recording chamber.

### *In vitro* Field Recording

Transverse and longitudinal hippocampal slices were transferred from the recovery beaker to the recording chamber, where they were submerged in aCSF (30 °C) with a flow rate of 2 ml/min. For the transverse slices, extracellular field potentials were recorded in the CA1 region using glass electrodes containing NaCl (3 M). A stimulating electrode in CA2 (Schaffer collateral pathway) was used to evoke field EPSPs (constant voltage, 100 μs duration, repeated at 30 sec intervals). For the longitudinal slices, recording and stimulating electrodes were placed at stratum oriens (S.O.) or stratum radiatum (S.R.) along the longitudinal axis to record extracellular field potentials and evoke field EPSPs. LTP was induced by two trains of tetanus stimuli (HFS; 100 Hz, 1 sec, repeated after a 30 sec interval). LTD experiments were performed with 4 protocols: 5 Hz LFS (900 stimuli during 3 min), 1 Hz LFS (900 stimuli during 15 min), 1 Hz paired-pulse LFS (50 ms paired-pulse interval, 900 pairs of stimuli during 15 min) and 50 μM DHPG, a group I mGluR agonist as a pharmacological induction of LTD. The slopes of the evoked fEPSPs were measured and expressed relative to the normalized preconditioning baseline. Experiments, in which changes in the fiber volley occurred, were discarded.

### 3D digital holography uncaging

The procedures for digital holographic photolysis have been described in detail elsewhere^18–20^. Unlike conventional uncaging techniques, the holographic method permits glutamate photolysis to be directed precisely and simultaneously at multiple sites and depths. Briefly, the holographic beam was brought into the optical axis of an upright fluorescence microscope (Olympus BX51) below the epi-fluorescence unit, with a long-pass dichroic mirror. The output beam of a 150 mW, 405 nm diode laser (CNI Laser) was expanded with a beam expander (3x) to fill the short axis of a reflective spatial light modulator (SLM) (LCOS Hamamatsu, model X10468–05). The SLM plane was projected onto the back aperture of the microscope objective through a telescope (L1, f1 = 750 mm; L2, f2 = 500 mm). The magnification of the telescope was chosen in order to match the SLM short axis with the diameter of the objective’s back aperture (Olympus, 60x, W 0.9NA). The undiffracted component (zero order spot) was removed by placing a small (<0.5 mm) anodized metal plate on antireflective coated glass plate at the focal plane of L1. The algorithm for the phase hologram calculation and calibration of the temporal spatial resolution have been described previously^18^. Fresh MNI-caged-L-glutamate (Tocris, Ellisville, MO) at final concentration in physiological solution was prepared each day. All agonists and antagonists were purchased from Sigma (St. Louis, MO) or Tocris (Ellisville, MO).

### Statistics

All data are shown as mean ± standard error (SE). An ANOVA of Fisher’s PLSD post hoc test was performed for between-group comparison, while a t-test was done for within-groups (significance, *P < 0.05; **P < 0.01).

## Results

### *In vivo* LTPs in the interlamellar CA1 network

In order to test whether the interlamellar CA1 connection shows long-term synaptic potentiation, recording and stimulus electrodes were placed along the longitudinal septal-temporal axis of hippocampus CA1 in anesthetized animals. Loci of recording (in a septal side) and stimulating (in a temporal side) electrodes were identified by lesion marks after completion of the electrophysiological recordings (Fig. 1a). 100 Hz HFS potentiated the synaptic responses in 10 unit recordings (Fig. 1b; 124 ± 4%, *t*-test, *t* = −4.79, *p* = 0.001). This result demonstrates that LTP exists in the interlamellar network of live animals.

### *In vitro* LTPs in interlamellar CA1 network

**Figure 2:**
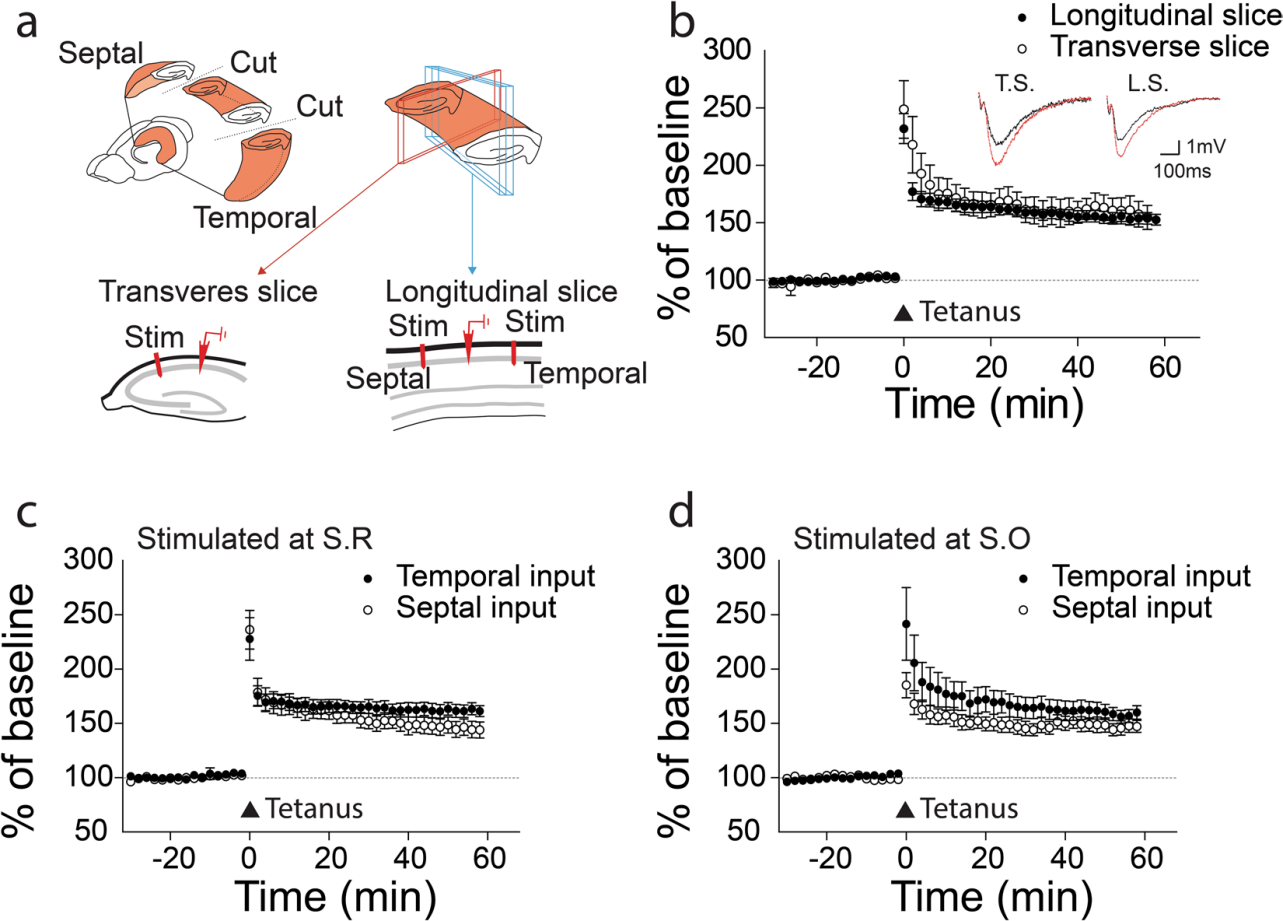
LTP in transverse slices and longitudinal slices. (**a**) A schematic drawing of transverse and longitudinal slices. (**b**) LTPs induced in transverse (n = 6) and longitudinal (n = 24) slices. Synaptic responses at S.R. (**c**) or S.O. (**d**) in longitudinal slices are potentiated right after tetanus stimulation with both temporal and septal inputs (S.R./temporal (n = 12, c), S.R./septal (n = 12, c), S.O./temporal (n = 10, d), S.O./septal (n = 9, d). The n stands for the number of slices. Error bars represent SE.

To further investigate direction- and layer-specific LTP within the interlamellar plane, longitudinal brain slices were prepared at a nearly perpendicular angle of the transverse slices according to our established protocol^12^. Synaptic responses were monitored with field recordings in both transverse and longitudinal slices (Fig. 2a). Longitudinal LTPs, induced by employing 100 Hz HFS in the area of the *stratum radiatum* (S.R.) of CA1 pyramidal neurons, were comparable to transverse LTPs (Fig. 2b, longitudinal LTP, 144 ± 5%, n = 24; transverse LTP, 159 ± 11%, n = 9). Next we tested, whether longitudinal LTPs are selective for direction or layer. Synaptic responses were induced by placing a stimulating electrode on either the temporal or the septal side of the *stratum radiatum* (SR). Electrical activation of both sides induced LTP without a significant difference between the two directions (Fig. 2c; temporal: 146 ± 5%, n = 12; septal: 143 ± 7%, n = 12; *P* > *0.1*). Similar to LTPs stimulated in S.R., LTPs observed in *stratum oriens* (SO) had no directional specificity, suggesting an absence of layer selectivity (Fig. 2d; temporal: 160 ± 6%, n = 10; septal: 147 ± 6%, n = 9; *P* > *0.1*).

### LTPs in CA1-CA1 pyramidal cells

**Figure 3:**
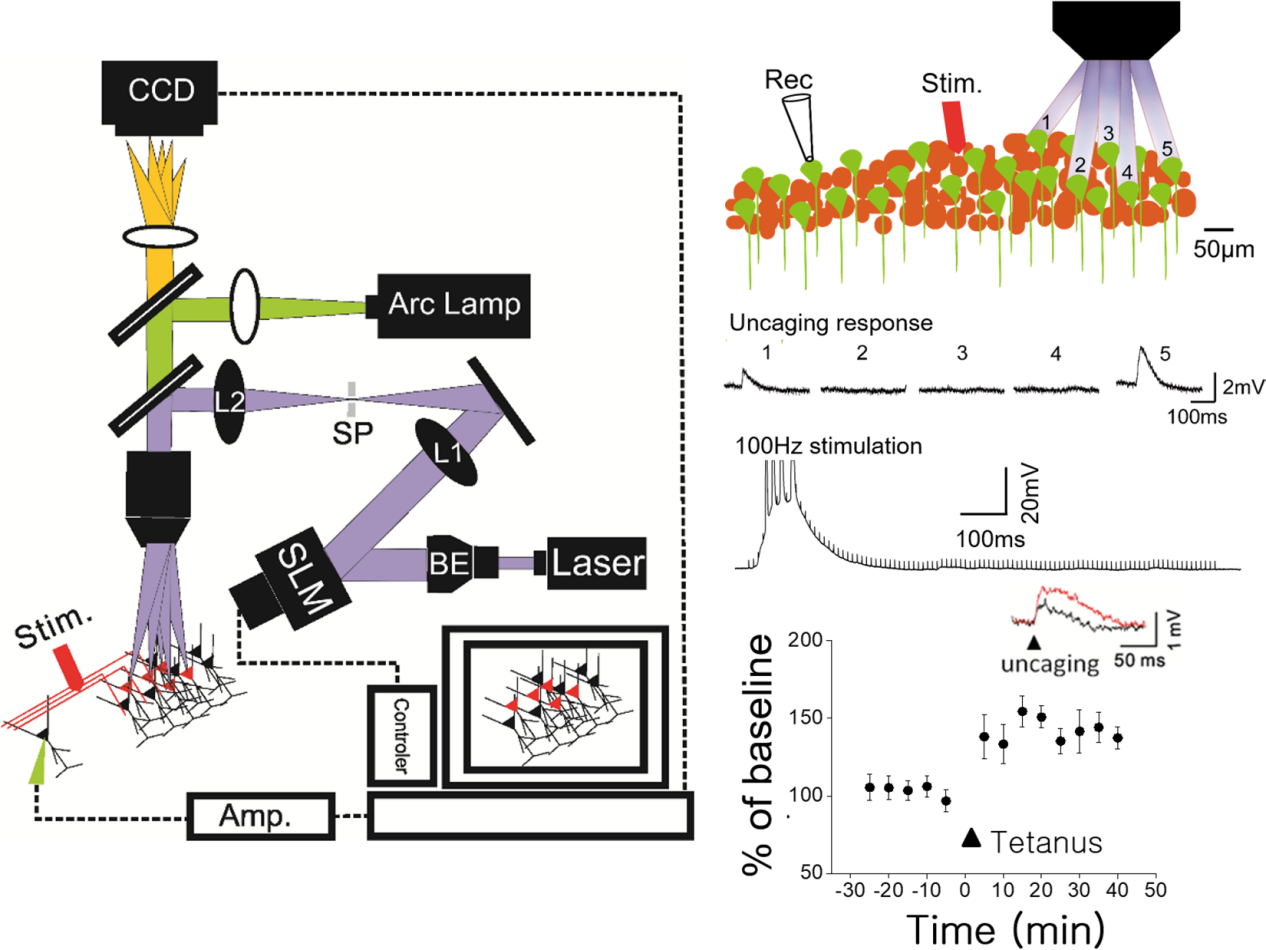
The long-term potentiation of the pyramidal to pyramidal connection. A schematic drawing demonstrating the 3D digital holographic photolysis setup (left). The holographic method permitted glutamate photolysis to be directed precisely at multiple sites and depths simultaneously. The light path for multiple glutamate uncaging was marked in purple, electrical stimulation in red. Photolysis for glutamate uncaging allowed us to identify pyramidal neurons connected to patched cells. In the example the pyramidal to pyramidal connection was identified by uncaging-induced synaptic responses at cell 1 and 5 but not at cells 2, 3, 4 (top right). HFS in longitudinal networks depolarized the postsynaptic neurons and potentiated uncaging-induced synaptic responses between individual pairs longitudinally connected CA1 pyramidal neurons (bottom right).

To confirm that LTPs in CA1-CA1 pyramidal cells is mediated by direct interaction between longitudinally connected excitatory neurons, we combined glutamate uncaging with a digital hologram system in longitudinal slices (Fig. 3a). After establishing whole-cell recording on a CA1 pyramidal neuron, we induced glutamate-mediated synaptic responses of the neuron by photostimulation of caged glutamate on neighboring pyramidal neurons. We monitored the uncaging-induced synaptic responses before and after the same 100 Hz tetanic stimulation we used for *in vivo* unit and *in vitro* field recordings. Electrical tetanic stimulation potentiated the uncaging-induced synaptic responses to 135.60 ± 11.03% (Fig. 3b; n = 10 neurons, *P* < *0.05*). These data indicate that excitatory CA1-CA1 synaptic connection mediates postsynaptic long-term synaptic plasticity.

### NMDA receptor-dependent LTPs in Interlamellar CA1 network

**Figure 4:**
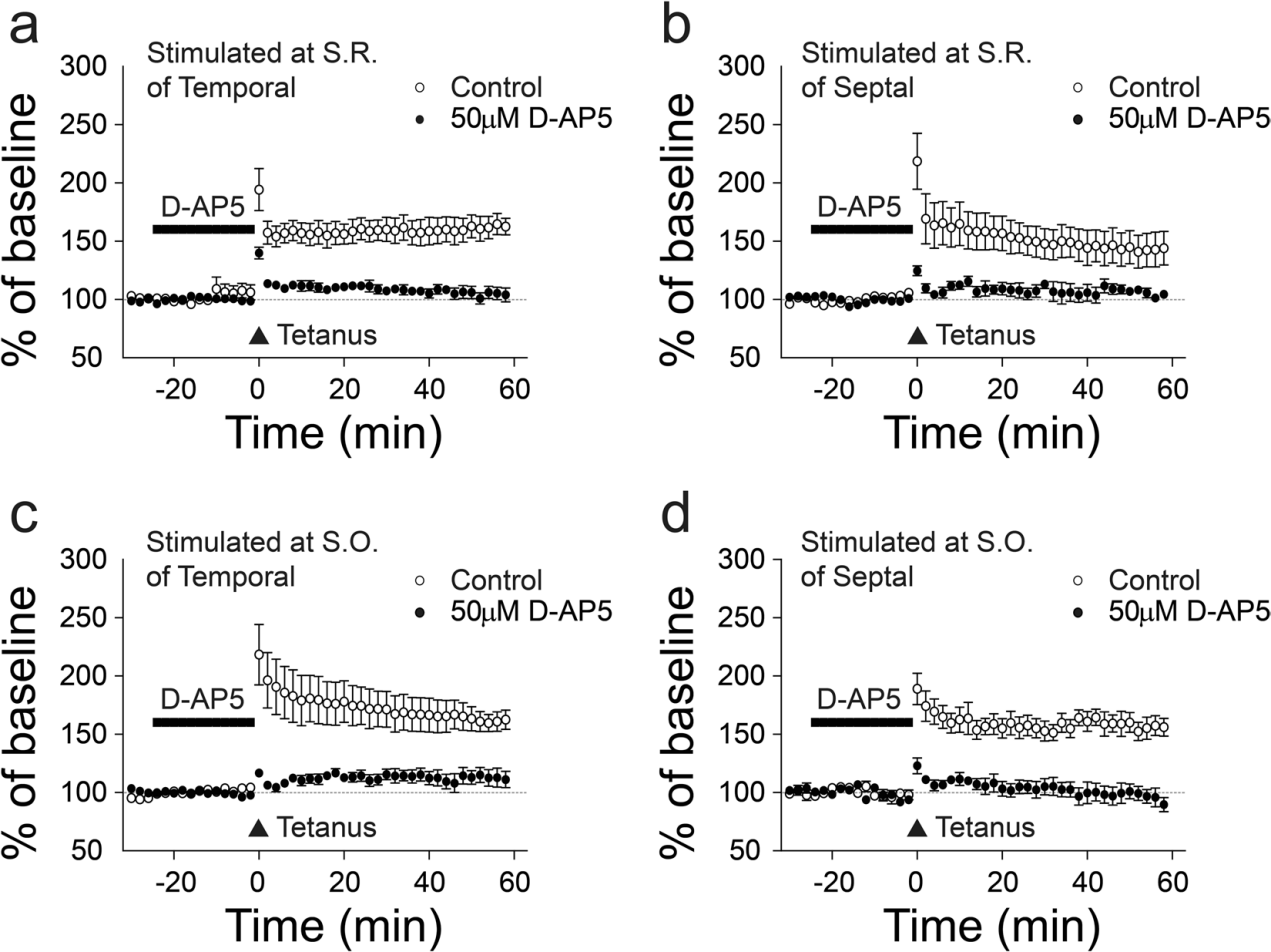
NMDAR-dependent LTP in longitudinal slices. (**a**,**b**) LTP induction in temporal and septal direction is blocked by 50 μM D-AP5 (temporal, n = 6, a) (septal, n = 5, b). (**c**,**d**) LTP induction in temporal and septal direction is also blocked by D-AP5. The n stands for slices. Error bars represent SE.

Next, we investigated the underlying mechanisms of the longitudinal LTP. In the presence of 50 μM D-AP5, an NMDAR antagonist, the enhancement of fEPSP after tetanus stimulation was reduced from 138 ± 7% (n = 6) to 113 ± 6% (n = 5) at the temporal and from 154 ± 14% (n = 6) to 111 ± 7% (n = 5) at the septal side of S.R. (Fig. 4a and b). Additionally, we investigated, whether LTP in S.O is NMDA dependent. Similar to the LTP reduction observed in S.R., application of D-AP5 attenuated LTPs from 170 ± 8% (n = 6) to 106 ± 7% (n = 6) at the temporal side and from 144 ± 8% (n = 5) to 97 ± 6% (n = 5) at the septal side (Fig. 4c and d) of S.O. These results confirm that NMDARs mediates LTPs predominantly without direction and layer selectivity.

### Absence of LTD in Interlamellar CA1 network

**Figure 5:**
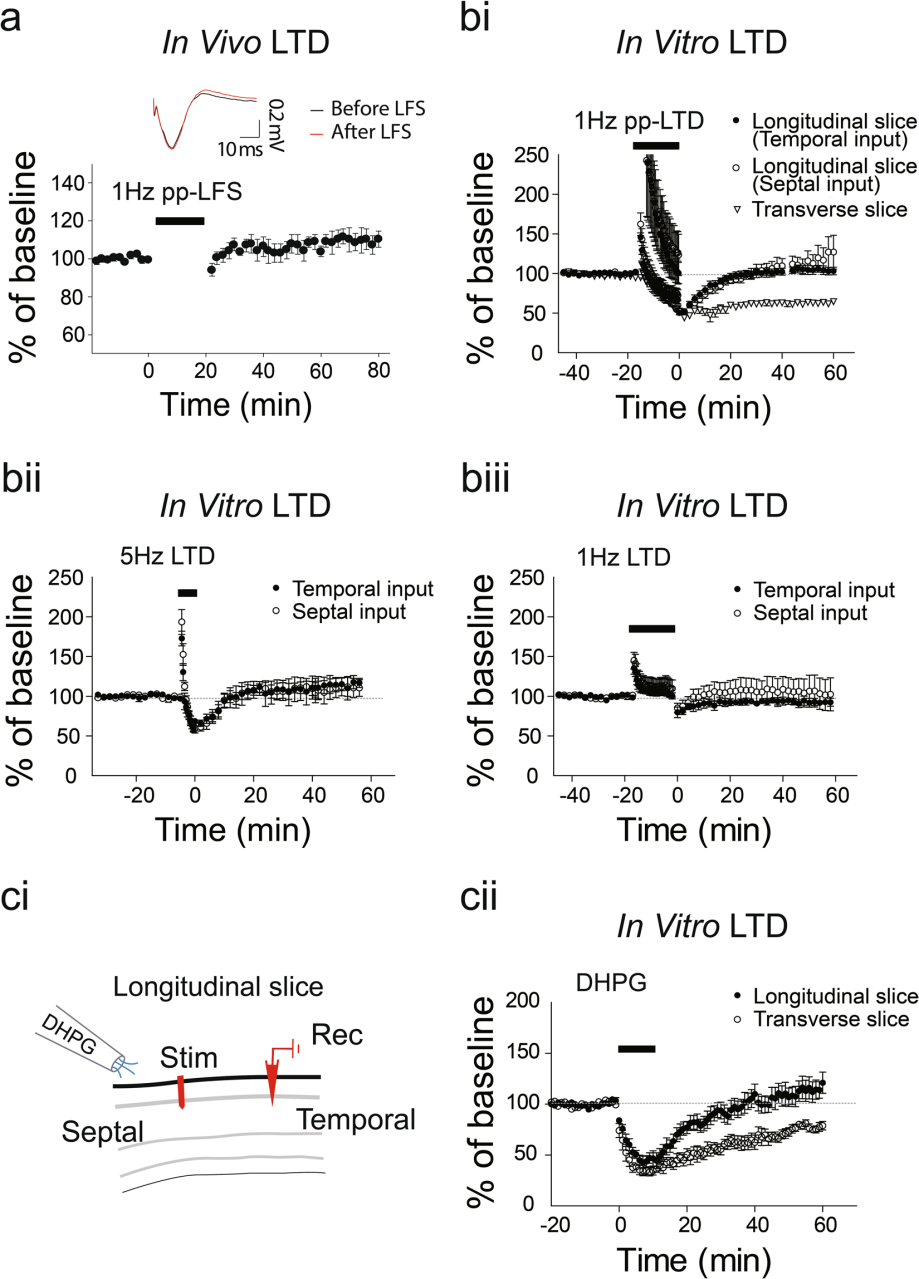
Absence of *in vivo* and *in vitro* LTD in Interlamellar CA1 network. (**a**) 1 Hz LFS does not induce *in vivo* LTD. (**b)** 1 Hz pp-LTP, 5 Hz LFS, and 1 Hz LFS do not produce LTD on either the temporal or septal sides, while LTD is induced by 1 Hz pp-LFS in transverse slices: temporal (n = 8), septal (n = 11) and transverse (n = 6) with 1 Hz pp-LFS; temporal (n = 3) and septal (n = 3) with 5 Hz LFS; temporal (n = 3) and septal (n = 3) with 1 Hz LFS. (**c**) Lack of mGluR-mediated LTD in a longitudinal slice. LTD is induced by application of DHPG for 10 minutes in transverse slices (n = 7) but not in longitudinal slices (n = 6). The n stands for slices. Error bars represent SE.

Low frequency stimulation of hippocampal networks, including the CA3-CA1 projection, induces LTD both *in vitro* and *in vivo*^7,21–28^. We investigated LTDs along the interlamellar CA1. Just as with the *in vivo* LTP induction protocol, we placed recording and stimulus electrodes along the longitudinal septal-temporal axis of hippocampus CA1 in anesthetized animals . However, 1 Hz pp-LFSs failed to induce LTD: no significant difference before and after 1 Hz pp-LFS of in the slope of fEPSPs was observed (fEPSP slope, 106 ± 1%, n = 12, *t*-test, *t* = −1.63, *p* > 0.1; Fig. 5a). To confirm the absence of LTD along the longitudinal axis, we recorded fEPSPs in brain slices under various known LTD protocols including 1 Hz pp-LFS, 5 Hz LFS, and 1 Hz LFS (Fig. 5bi)^7,29–33^. Consistent with our *in vivo* data, *in vitro* LTD was not observed under these three LTD protocols (Fig. 5b, *1 Hz pp-LFS*, temporal: 104 ± 5%, n = 8, *P* > *0.1*; septal: 121 ± 17%, n = 11, *P* > *0.1; transverse: 63* ± 3%, n = 6; bii, *5 Hz LFS*, temporal: 115 ± 9%, n = 3, *P* > *0.1*; septal: 111 ± 12%, n = 3, *P* > *0.1*; biii; *1 Hz LFS* temporal: 92 ± 44%, n = 3, *P* > *0.1*; septal: 104 ± 20%, n = 3, *P* > *0.1*). This demonstrates that the longitudinal network does not manifest electrically-induced LTDs. Also, we wondered whether the longitudinal network is involved in mGluR-mediated LTDs. The mechanism of mGluR-mediated LTDs were known to be different on that of NMDAR-mediated LTDs; they are tightly associated with endocytosis of synaptic AMPA receptors through Arc/arg 3.1 signaling^30,34,35^. Here, we tested the mGluR-mediated LTDs in both transverse and longitudinal networks. DHPG, a group I mGluR agonist, induced LTDs in transverse networks (Fig. 5c; 77 ± 3.5%, n = 7), but not in longitudinal network (113 ± 9.8%, n = 6). These results suggest that the longitudinal network is resistant to LTD induction, implicating unique synaptic properties of interlamellar connections compared to lamellar synapses.

## Discussion

The CA1 region on the transverse axis of hippocampus is a common model for studying the cellular basis of cognitive functions. Research into CA1 function has produced an extraordinarily rich body of knowledge on hippocampus physiology^3^. Yet, it has remained unclear, whether CA1 efferent fibers project along the long axis of hippocampus^36,37^ and that being the case, whether they play a role in synaptic plasticity. Here we show that the longitudinal network exhibits robust LTP but no significant LTD under many known LTD induction protocols.

The synaptic potentiation of interlamellar CA1 network could contribute to hippocampus-related memory. Hampson *et al*.^8^ carried out multi-electrode recordings in the hippocampus of rats that were performing a short-term memory task, they observed clusters of cells responding to both place and “phase” cues in orderly organized segments along the longitudinal axis^8^. Recently, Long *et al*. (2015) reported that hippocampal theta signals, which are known to be involved in sensory integration, attention and memory, are synchronized across the septotemporal axis of CA1. Also, hippocampal sharp wave and ripple (SPW/R) signals, which play an essential role in the formation and consolidation of episodic memory, are known to be generated and propagated along the CA1 longitudinal connectivity^38,39^. Consistent with these findings, the longitudinal projection may serve as an anatomical substrate of this behaviorally relevant signal processing^12^.

The mechanism regarding the absence of LTD in longitudinal network is not clear. It has been known that LTD requires the involvement of both presynaptic (such as CB1 receptors and calcineurin) and postsynaptic mechanisms (such as NMDA receptors, mGluRs, phospholipase C, and endocannabinoid synthesis)^21,40,41^. For example, when mGluRs and CB1 receptors are blocked, this leads to a suppression of lamellar LTD *in vitro*^42,43^ and *in vivo*^44,45^. We speculate that the absence of LTD in the interlamellar connection is due to the absence or lack of these LTD inducing machineries. Besides the molecular mechanisms, the absence of LTDs can be explained by structural factors such as an axon properties (thick axonal diameter and low varicosity density in the transverse projection vs. thin axonal diameter and high varicosity density in the longitudinal projection^12^. For now, our finding just showed favorable LTPs but not LTDs in the longitudinal projection, presumably being involved in strong connectivity and hyperexcitability. Longitudinally oriented synaptic networks were identified as potential foci in patients with hippocampal epilepsy. Seizure activity was found to synchronize along the entire length of hippocampus via the longitudinal projection^46^. Transverse cuts that severed the longitudinal pathway in human hippocampus are an effective treatment to prevent hippocampal seizures from spreading^46,47^. Both the length and number of axon collaterals of CA1 neurons are increased in rats that experience chronic limbic seizures following kainic acid or pilocarpine application^48,49^. These results implicate that epileptiform activity may travel along the longitudinal axis of hippocampus. Based on ours and other studies, we speculate that the thin axonal diameter (probably having high impedance) and high varicosity density can be favorable for fast signal spread in multiple spots simultaneously. These axonal properties can promote a prompt spread of epileptiform activity and enhance memory function. However, we await further researches concerning favorable LTPs but not LTDs in the longitudinal networks. In conclusion, we pose that the CA1 longitudinal network is a promising candidate to investigate memory-related information processing and brain diseases.

## References

[1] Amaral DG, Witter MP (1989). The three-dimensional organization of the hippocampal formation: a review of anatomical data. Neuroscience.

[2] Tulving E, Markowitsch HJ (1998). Episodic and declarative memory: role of the hippocampus. Hippocampus.

[3] Bliss TV, Collingridge GL (1993). A synaptic model of memory: long-term potentiation in the hippocampus. Nature.

[4] Andersen P, Bland BH, Dudar JD (1973). Organization of the hippocampal output. Exp Brain Res.

[5] Andersen P, Bliss TV, Lomo T, Olsen LI, Skrede KK (1969). Lamellar organization of hippocampal excitatory pathways. Acta Physiol Scand.

[6] LeGates TA (2012). Aberrant light directly impairs mood and learning through melanopsin-expressing neurons. Nature.

[7] Yang S (2013). Integrity of mGluR-LTD in the associative/commissural inputs to CA3 correlates with successful aging in rats. The Journal of neuroscience: the official journal of the Society for Neuroscience.

[8] Hampson RE, Simeral JD, Deadwyler SA (1999). Distribution of spatial and nonspatial information in dorsal hippocampus. Nature.

[9] Alger BE, Teyler TJ (1977). A monosynaptic fiber track studied *in vitro*: evidence of a hippocampal CA1 associational system?. Brain Res Bull.

[10] Deuchars J, Thomson AM (1996). CA1 pyramid-pyramid connections in rat hippocampus *in vitro*: dual intracellular recordings with biocytin filling. Neuroscience.

[11] Shao LR, Dudek FE (2004). Increased excitatory synaptic activity and local connectivity of hippocampal CA1 pyramidal cells in rats with kainate-induced epilepsy. Journal of neurophysiology.

[12] Yang S (2014). Interlamellar CA1 network in the hippocampus. Proc Natl Acad Sci USA.

[13] Malenka RC, Bear MF (2004). LTP and LTD: an embarrassment of riches. Neuron.

[14] Lynch MA (2004). Long-term potentiation and memory. Physiol Rev.

[15] Kumar A (2011). Long-Term Potentiation at CA3-CA1 Hippocampal Synapses with Special Emphasis on Aging, Disease, and Stress. Front Aging Neurosci.

[16] Bliss TV, Lomo T (1973). Long-lasting potentiation of synaptic transmission in the dentate area of the anaesthetized rabbit following stimulation of the perforant path. J Physiol.

[17] Jo J (2011). Aβ(1–42) inhibition of LTP is mediated by a signaling pathway involving caspase-3, Akt1 and GSK-3β. Nat Neurosci.

[18] Yang S (2011). Three-dimensional holographic photostimulation of the dendritic arbor. J Neural Eng.

[19] Yang S, Tang CM, Yang S (2015). The Shaping of Two Distinct Dendritic Spikes by A-Type Voltage-Gated K(+) Channels. Frontiers in cellular neuroscience.

[20] Yang S, Santos MD, Tang CM, Kim JG, Yang S (2016). A Postsynaptic Role for Short-Term Neuronal Facilitation in Dendritic Spines. Frontiers in cellular neuroscience.

[21] Kemp A, Manahan-Vaughan D (2004). Hippocampal long-term depression and long-term potentiation encode different aspects of novelty acquisition. Proc Natl Acad Sci USA.

[22] Hagena H, Manahan-Vaughan D (2010). Frequency facilitation at mossy fiber-CA3 synapses of freely behaving rats contributes to the induction of persistent LTD via an adenosine-A1 receptor-regulated mechanism. Cereb Cortex.

[23] Kimura T (2014). Microtubule-associated protein tau is essential for long-term depression in the hippocampus. Philos Trans R Soc Lond B Biol Sci.

[24] Aksoy-Aksel A, Manahan-Vaughan D (2013). The temporoammonic input to the hippocampal CA1 region displays distinctly different synaptic plasticity compared to the Schaffer collateral input *in vivo*: significance for synaptic information processing. Front Synaptic Neurosci.

[25] Collingridge GL, Peineau S, Howland JG, Wang YT (2010). Long-term depression in the CNS. Nat Rev Neurosci.

[26] Lüscher, C. & Malenka, R. C. NMDA receptor-dependent long-term potentiation and long-term depression (LTP/LTD). *Cold Spring Harb Perspect Biol* **4**, 10.1101/cshperspect.a005710 (2012). 10.1101/cshperspect.a005710 PMC3367554 22510460

[27] Jo J (2008). Metabotropic glutamate receptor-mediated LTD involves two interacting Ca(2+) sensors, NCS-1 and PICK1. Neuron.

[28] Jo J (2010). Muscarinic receptors induce LTD of NMDAR EPSCs via a mechanism involving hippocalcin, AP2 and PSD-95. Nat Neurosci.

[29] Fan W, Ster J, Gerber U (2010). Activation conditions for the induction of metabotropic glutamate receptor-dependent long-term depression in hippocampal CA1 pyramidal cells. The Journal of neuroscience: the official journal of the Society for Neuroscience.

[30] Huber KM, Kayser MS, Bear MF (2000). Role for rapid dendritic protein synthesis in hippocampal mGluR-dependent long-term depression. Science.

[31] Kemp N, Bashir ZI (1997). NMDA receptor-dependent and -independent long-term depression in the CA1 region of the adult rat hippocampus *in vitro*. Neuropharmacology.

[32] Jo J (2006). Experience-dependent modification of mechanisms of long-term depression. Nature neuroscience.

[33] Bear MF, Abraham WC (1996). Long-term depression in hippocampus. Annual review of neuroscience.

[34] Luscher, C. & Huber, K. M. Group 1 mGluR-dependent synaptic long-term depression: mechanisms and implications for circuitry and disease. *Neuron* **65**, 445–459, 10.1016/j.neuron.2010.01.016. 10.1016/j.neuron.2010.01.016 PMC2841961 20188650

[35] Park S (2008). Elongation factor 2 and fragile X mental retardation protein control the dynamic translation of Arc/Arg3.1 essential for mGluR-LTD. Neuron.

[36] Amaral DG, Dolorfo C, Alvarez-Royo P (1991). Organization of CA1 projections to the subiculum: a PHA-L analysis in the rat. Hippocampus.

[37] Tamamaki N, Nojyo Y (1990). Disposition of the slab-like modules formed by axon branches originating from single CA1 pyramidal neurons in the rat hippocampus. J Comp Neurol.

[38] Jahnke S, Timme M, Memmesheimer RM (2015). A Unified Dynamic Model for Learning, Replay, and Sharp-Wave/Ripples. The Journal of neuroscience: the official journal of the Society for Neuroscience.

[39] Hulse BK, Moreaux LC, Lubenov EV, Siapas AG (2016). Membrane Potential Dynamics of CA1 Pyramidal Neurons during Hippocampal Ripples in Awake Mice. Neuron.

[40] Andrade-Talavera Y, Duque-Feria P, Paulsen O, Rodríguez-Moreno A (2016). Presynaptic Spike Timing-Dependent Long-Term Depression in the Mouse Hippocampus. Cereb Cortex.

[41] Kemp A, Manahan-Vaughan D (2008). Beta-adrenoreceptors comprise a critical element in learning-facilitated long-term plasticity. Cereb Cortex.

[42] Neyman S, Manahan-Vaughan D (2008). Metabotropic glutamate receptor 1 (mGluR1) and 5 (mGluR5) regulate late phases of LTP and LTD in the hippocampal CA1 region *in vitro*. Eur J Neurosci.

[43] Mukherjee S, Manahan-Vaughan D (2013). Role of metabotropic glutamate receptors in persistent forms of hippocampal plasticity and learning. Neuropharmacology.

[44] Naie K, Manahan-Vaughan D (2004). Regulation by metabotropic glutamate receptor 5 of LTP in the dentate gyrus of freely moving rats: relevance for learning and memory formation. Cereb Cortex.

[45] Manahan-Vaughan D, Braunewell KH (2005). The metabotropic glutamate receptor, mGluR5, is a key determinant of good and bad spatial learning performance and hippocampal synaptic plasticity. Cereb Cortex.

[46] Umeoka SC, Luders HO, Turnbull JP, Koubeissi MZ, Maciunas RJ (2012). Requirement of longitudinal synchrony of epileptiform discharges in the hippocampus for seizure generation: a pilot study. J Neurosurg.

[47] Shimizu H, Kawai K, Sunaga S, Sugano H, Yamada T (2006). Hippocampal transection for treatment of left temporal lobe epilepsy with preservation of verbal memory. J Clin Neurosci.

[48] Perez Y, Morin F, Beaulieu C, Lacaille JC (1996). Axonal sprouting of CA1 pyramidal cells in hyperexcitable hippocampal slices of kainate-treated rats. Eur J Neurosci.

[49] Esclapez M, Hirsch JC, Ben-Ari Y, Bernard C (1999). Newly formed excitatory pathways provide a substrate for hyperexcitability in experimental temporal lobe epilepsy. J Comp Neurol.

